# Reducing medical claims cost to Ghana’s National Health Insurance scheme: a cross-sectional comparative assessment of the paper- and electronic-based claims reviews

**DOI:** 10.1186/s12913-017-2054-1

**Published:** 2017-02-06

**Authors:** Eric Nsiah-Boateng, Francis Asenso-Boadi, Lydia Dsane-Selby, Francis-Xavier Andoh-Adjei, Nathaniel Otoo, Patricia Akweongo, Moses Aikins

**Affiliations:** 1 National Health Insurance Authority, Accra, Ghana; 2 School of Public Health, University of Ghana, Accra, Ghana

**Keywords:** Medical claims review, Claims adjustment, National Health Insurance Scheme, Ghana

## Abstract

**Background:**

A robust medical claims review system is crucial for addressing fraud and abuse and ensuring financial viability of health insurance organisations. This paper assesses claims adjustment rate of the paper- and electronic-based claims reviews of the National Health Insurance Scheme (NHIS) in Ghana.

**Methods:**

The study was a cross-sectional comparative assessment of paper- and electronic-based claims reviews of the NHIS. Medical claims of subscribers for the year, 2014 were requested from the claims directorate and analysed. Proportions of claims adjusted by the paper- and electronic-based claims reviews were determined for each type of healthcare facility. Bivariate analyses were also conducted to test for differences in claims adjustments between healthcare facility types, and between the two claims reviews.

**Results:**

The electronic-based review made overall adjustment of 17.0% from GHS10.09 million (USD2.64 m) claims cost whilst the paper-based review adjusted 4.9% from a total of GHS57.50 million (USD15.09 m) claims cost received, and the difference was significant (*p* < 0.001). However, there were no significant differences in claims cost adjustment rate between healthcare facility types by the electronic-based (*p* = 0.0656) and by the paper-based reviews (*p* = 0.6484).

**Conclusions:**

The electronic-based review adjusted significantly higher claims cost than the paper-based claims review. Scaling up the electronic-based review to cover claims from all accredited care providers could reduce spurious claims cost to the scheme and ensure long term financial sustainability.

**Electronic supplementary material:**

The online version of this article (doi:10.1186/s12913-017-2054-1) contains supplementary material, which is available to authorized users.

## Background

Many developing countries particularly those in Sub-Saharan Africa are at different stages of implementing social health insurance schemes aimed at providing access to healthcare for the citizenry [1–3]. Evidence; however, abounds that sustainability of this model of healthcare financing depends on efficient claims management system to detect errors, abuse, and fraud [4]. Healthcare fraud and abuse are major concerns facing many healthcare systems particularly health insurance organisations. It is estimated that fraud and abuse cost up to 10% of total healthcare expenditure in countries worldwide [5, 6] and about $700 billion in the United States healthcare system [7].

In the health insurance industry, medical claims review is mostly outsourced to independent reviewers with highly specialised domain knowledge, due to its time-consuming process and great attention to details [8]. In state-owned health insurance systems, for example, the South Korean and Japanese systems, there are established independent claims review institutions for overseeing claims review and reimbursement [9–11]. Other health insurance organisations are increasingly using data mining techniques, a combination of automated methods and statistical knowledge in an emerging interdisciplinary branch of science called Knowledge Discovery from Databases (KDD), to detect medical claims fraud and abuse [12–14].

Studies show that a robust medical claims review system is crucial for addressing fraud and abuse, and ensuring cost-containment and long-term financial sustainability of health insurance organisations [9, 12, 15]. Besides, an efficient claims review and reimbursement process is an important aspect of quality of care since it contributes to early settlement of claims thereby incentivizing healthcare providers to deliver continuous and quality of care service to the insured. It also provides an overview of patterns of care, which influence policy directives for healthcare practitioners.

In 2003, Ghana introduced National Health Insurance Scheme (NHIS) through an Act of Parliament, National Health Insurance Act 650 (now Act 852), to replace out-of-pocket payment system popularly referred to as “cash and carry” [16–18]. According to Ministry of Health, the “cash and carry” system in the 1990s led to a widened gap of access to healthcare, poor health and avoidable deaths. It was estimated that out of the 18% of the population who needed healthcare at any given time, only one-fifth of them could afford it [19].

Since its full implementation in 2005, the NHIS has made tremendous strides in enrolment, provision of financial access to healthcare, and revenue to public and private healthcare providers [3, 20]. Currently, there are 10 regional offices, 159 district offices and 5 registration centres of the NHIS across the country [21]. The district and regional offices are mandated to enrol people into the scheme and collect premium as well. According to an unpublished 2014 annual report of the National Health Insurance Authority (NHIA), there are 10.5 million subscribers, representing 39% of the national population. The report also shows that 29.64 million outpatient utilization claims and 1.62 million inpatient utilization claims were reviewed, and a total amount of GHS968.48 million (USD254.19 million) was paid. This payment contributed about 80% of healthcare providers’ internally generated fund.

Over the past few years, NHIA has undertaken a number of reforms to make the NHIS more efficient, attractive, and sustainable in the long-term. Among these reforms are centralization of the NHIS claims review and reimbursement process and establishment of electronic claims review system. Presently, the NHIS has four Claims Processing Centres (CPC) in Accra, Cape Coast, Kumasi and Tamale but they serve all regions of the country. Together, these CPCs handle claims from 1,027 healthcare providers out of a total of 4004 [20]. Claims from the remaining healthcare providers are reviewed at some of the district offices across the country.

There have been a number of studies on the NHIS since its inception; however, majority of them focused on membership coverage, access to health services, and financial sustainability. A study that looked at claims management of the scheme was limited to claims management process, reimbursement rate, and value of rejected claims in two district offices in the Upper East Region [15]. Our study sought to go beyond claims review process to determine the capacity of the paper-based and electronic-based claims review systems to detect spurious claims and reduce cost to the NHIS. It is hoped that assessing performance of the claims review system, following recent reforms undertaken to improve efficiency and reduce claims cost, would help provide insight into claims management system of the scheme.

### Overview of the NHIS claims review and reimbursement system

**Fig. 1:**
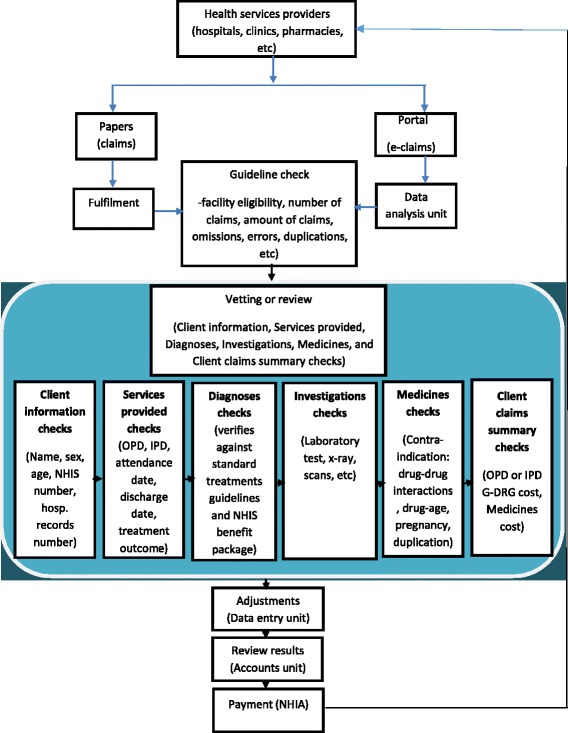
Flow chart of NHIA claims review and reimbursement process

Under the NHIS claims management system, the healthcare providers submit claims either in a paper form or through the electronic portal to the Accra CPC for review and payment (Fig. 1). The “fulfilment” stage is the first point of receipt of the paper claims, where the claims are checked for appropriateness in terms of the number and amount with what is stated in the cover letter and the summary sheet that accompanied the claims. The eligibility of the provider to provide services to the NHIS subscribers is also verified at this stage. Depending on the number of claims with problems, the fulfilment unit either writes a report of its initial review and sends it together with the claims to the claims reviewers for a complete review or returns the problematic claims to the health services providers for correction and resubmission. The second stage of the paper-based claims review process is the “vetting” stage, where checks are conducted on the various sections of the claims form such as client information, services provided, and medical procedures performed. Other sections that are also reviewed include diagnoses, investigations (laboratories, imaging), and medicines supplied.

The client information section is the first section of the “vetting stage”, where review is conducted on the biodata, insurance member number, and hospital record number of the insured. Inadequate information on the client would lead to partial or complete rejection of the claim, depending on the extent of the problem. In the services provided section, review is conducted on the type of service provided, the attendance date, and if applicable, the discharge date and outcome of the treatment. In addition, if there were any other medical procedures performed, for example, surgical procedures, then the appropriateness of the procedure is verified. Review of the Diagnoses section involves checks on age of the insured patient, type of service provided, and level of healthcare provider, as well as the Ghana Diagnosis Related Groupings (G-DRG) code. A claim is rejected when it is found that the diagnosis or disease condition stated is clinically inappropriate for the insured patient due to factors including sex and age. A claim is also rejected if the care provider did not have the mandate to provide that service based on the NHIS accreditation guidelines. Review of the investigations section involves checks on the type of laboratory test conducted or diagnostic performed. In the Medicines section, contraindication issues such as prescribing levels, drug-age, multiple use of same class of drugs together, and prescription of unsafe quantities are checked. Other areas that are also checked include duplication, price codes, quantities of medicines, and the link between diagnosis and medicines. The client claim summary is the last section of the claims vetting stage, where the costs of services provided are summarized. In this section, checks are performed on the total amount for the non-medicine and medicine services against the G-DRG codes. After review of all the sections, claims that meet the review guidelines are approved and paid whilst those that did not are partially or completely rejected.

## Methods

This study was a cross-sectional comparative assessment of the paper- and electronic-based claims reviews of the NHIA. Medical claims of NHIS subscribers submitted for the period January to December, 2014 were requested from the claims directorate of the NHIA. This period contained reliable data of claims that had been reviewed and reimbursed following establishment of the electronic claims review system. The claims comprised of both physical forms and electronic data which contained subscribers’ demographic information such as name, sex, age, and insurance member number. Other information included date of attendance, diagnosis, cost of service, cost of medicines, and names of healthcare providers. The claims were submitted by accredited public, private, and faith-based healthcare facilities in the ten administrative regions of the country. These facilities included community pharmacies, community diagnostic centres, health centres and maternity homes. Others were clinics, district hospitals, regional hospitals, and national level referral hospitals.

### Data analysis

Quantitative analysis of the claims data focused on adjustment rate of the claims review system, defined as the ratio of erroneous claims cost to the total claims cost, multiplied by 100%. This analysis was conducted for each type of healthcare facility (pharmacy, health centre, clinic, polyclinic, district hospital, regional hospital, teaching/tertiary hospital), and the results presented in tables. The unit of analysis was the number of submissions made by each healthcare facility to the Accra CPC.

### Statistical analysis

Analysis of variance (ANOVA) general linear model (GLM) was conducted to test for differences in adjustment rates between the healthcare facility types for both paper- and electronic-based reviews. Two independent samples *t*-test was also performed to test for differences in adjustment rate between the paper- and electronic-based review systems. Since the outcome variable (adjustment rate) was in percentage, and the data were not normally distributed, they were transformed into normal curve equivalence (NCE) scores with a mean of 50 and standard deviation of 21.06 [22] before conducting the ANOVA test. A threshold of *p* < 0.05 was set for statistical significance, and Stata version 13 software was used for the analysis.

## Results

### Characteristics of the medical claims data

A total of 2.1 million number of claims with a cost of GHS67.60 million (USD17.74 m) were submitted to the Accra CPC for review and payment (Table 1). These claims were submitted by 173 healthcare providers for NHIS subscribers who utilized healthcare in the year 2014. The health centres and clinics made the highest number of submissions (596); however, the polyclinics and district hospitals submitted the highest number of claims (1.2million) and cost of claims (GHS36.80 million), which represent 56 and 54% of the total number and cost of claims, respectively.

**Table 1:** Summary of the medical claims data

Healthcare facility type	No. of facility	No. of submission	No. of claims submitted (%)	Cost of claims Submitted in GHS (%)
Pharmacies	19	127 (10.7)	84,904 (4.1)	2,563,466.00 (3.8)
Health centres & Clinics*	78	596 (50.4)	438,123 (21.1)	11,271,330.00 (16.7)
Polyclinics & District hospitals	67	404 (34.2)	1,157,698 (55.9)	36,802,348.00 (54.4)
Regional hospitals	6	27 (2.3)	206,673 (10.0)	7,307,068.00 (10.8)
Tertiary/Teaching hospitals	3	28 (2.4)	184,725 (8.9)	9,646,613.00 (14.3)
Total	173	1,182 (100.00)	2,072,123 (100.0)	67,590,825.00 (100.0)

GHS: Ghana Cedis; Currency exchange rate of USD1.00:GHS3.81; *includes diagnostic centres and maternity homes

### Claims cost adjustment rate of the paper-based claims review

The paper-based claims review system reviewed a total of GHS57.50 million (USD15.09 m) claims cost, and adjusted GHS2.81 million (4.9%) (Table 2). The proportions of claims adjusted by type of healthcare facility shows that the Regional hospitals recorded the highest adjustment rate of 6.3%; although, they submitted a lower claims cost compared to the health centres and clinics, polyclinics and district hospitals, and tertiary/teaching hospitals (Table 2). The polyclinics and district hospitals submitted the highest claims cost but recorded relatively lower adjustment rate of 5.1%. Result of the ANOVA GLM revealed that there was no statistically significant difference in adjustment rate between the healthcare facility types, *F*(4, 794) = 0.62, *p* = 0.65 (see Additional file 1).

**Table 2:** Paper-based claims review adjustment by healthcare facility type

Healthcare facility type	Claims cost received (GHS)	Claims cost adjusted (GHS)	Adjustment rate (%)
Pharmacies	2,563,466.00	71,911.00	2.8
Health centres & clinics	11,271,330.00	650,707.00	5.8
Polyclinics & District hospitals	29,655,401.00	1,506,261.00	5.1
Regional hospitals	4,621,898.00	291,470.00	6.3
Tertiary/Teaching hospitals	9,387,994.00	291,029.00	3.1
Total	57,500,089.00	2,811,459.00	4.9

*GHS* Ghana Cedis, Currency exchange rate of USD1.00:GHS3.81

### Claims cost adjustment rate of the electronic-based claims review

About GHS10.09 million (USD2.64 m) total claims cost was submitted to the Accra CPC for electronic-based review and payment, of which 17.0% was adjusted (Table 3). The tertiary/teaching hospitals submitted the lowest claims cost but had the highest adjustment rate of 24.3%. On the other hand, the district hospitals submitted the highest claims cost but recorded relatively lower adjustment rate of 17.9%. Adjustments made from claims of other healthcare facilities were less than 14.0%. Result of the bivariate analysis showed that there was no statistically significant difference in cost adjustment rate between the healthcare facility types, *F*(2, 82) = 2.82, *p* = 0.06 (see Additional file 2).

**Table 3:** Electronic-based claims review adjustment by healthcare facility type

Healthcare facility type	Claims cost received (GHS)	Claims cost adjusted (GHS)	Adjustment rate (%)
District hospitals	7,146,947.00	1,281,061.00	17.9
Regional hospitals	2,685,170.00	374,533.00	13.9
Tertiary/Teaching hospitals	258,619.00	62,796.00	24.3
Total	10,090,736.00	1,718,390.00	17.0

*GHS* Ghana Cedis, Currency exchange rate of USD1.00:GHS3.81

### Difference in cost adjustment rate between the paper- and electronic-based reviews

Two independent samples *t*-test conducted showed that adjustment rate by the electronic-based review was statistically significantly higher than that by the paper-based review, *t*(882) = −11.67, *p* < 0.001 (see Additional file 3).

## Discussion

This study sought to assess claims cost adjustment rate of the paper- and electronic-based claims reviews of the NHIS in Ghana. The paper-based review handles claims from the community level to the national level healthcare facilities whilst the electronic-based review handles claims from selected district, regional, and national level hospitals. Findings of the study revealed that there is significant difference in cost adjustment rate between the paper- and electronic-based reviews whilst healthcare facility types showed no significant difference, as elaborated below.

Overall adjustment rate of the electronic-based review was significantly higher than the one recorded from the paper-based claims review. This result is expected since the electronic-based review handles claims from the higher level of care, i.e., the hospitals, which are mostly inpatient and requires strict adherence to claims rules and regulations. In addition, the electronic-based review has in-built clinical rules to flag problematic claims that otherwise would have gone through the paper-based review undetected. For example, claims with issues of duplication, membership ineligibility, contraindication; that is, drug-drug and drug-age interactions, are more likely to pass through the paper-based claims review undetected compared to the electronic-based review. The policy implication is that the electronic-based claims review could reduce substantial cost to the NHIS and ensure long term sustainability.

Reason for the overall low adjustment rate by the paper-based claims review might be that majority of the healthcare providers now understand the claims preparation process based on their previous experience with the application of the G-DRG codes and tariff, as well as the medicine price list. Many of the care providers have been delivering services to the NHIS subscribers for over 10 years; as a result, they might have learned from their previous mistakes in claims preparations through meetings and training organised by the NHIA. Another plausible explanation for the low rate of adjustment is that healthcare providers themselves pre-screen their claims for errors before submitting to the CPC; an exercise that could eliminate omissions and duplications. The finding supports a study by Nsiah-Boateng et al. [23]; however, it contradicts a similar study, where the proportions of claims cost adjusted were 11 and 14% for two NHIA district offices in the Upper East region of Ghana [15].

Cost adjustments by type of healthcare facility shows that there were high rates of adjustments from the regional hospital claims by the paper-based review, a result inconsistent with a study by Park et al. [9]. The highest adjustment made from the comparatively small amount of the regional hospitals claims suggests that these providers may have limited understanding of the NHIS claims preparations and procedures. It might also indicate an element of fraud and abuse on the side of the care providers. The same can be said of the health centres and clinics, which submitted relatively small amount of claims but had high proportion of adjustments from their claims. This result; however, is similar to findings from an earlier study, where the lower level facilities recorded higher adjustments from their claims [9]. What makes the relatively high adjustments from the health centres and clinics claims more curious is that these facilities do not provide inpatient services, which require more detailed information and strict compliance to claims preparation guidelines. Rules for the preparation of outpatient claims are simpler and straightforward; therefore, further study would be needed to establish reasons for this high adjustments from health centres and clinics claims.

The positive sign; though, is the high number of claims from these healthcare facilities (health centres, clinics, polyclinics, district hospitals), which indicates that majority of the subscribers are utilizing more services at the primary level of the healthcare system, where cost is comparatively lower. This is in line with findings from a study on the Korean health insurance system, where patients utilized more services from the clinics and small hospitals compared to secondary and tertiary hospitals [9]. The result also indicates some level of acceptability of the NHIA’s gate-keeper system, where subscribers are restricted to use lower level healthcare facilities as the first point of call whenever they fall sick. This measure is aimed at preventing abuse in the use of healthcare at the secondary and tertiary healthcare facilities, where the cost is relatively higher. The increasing number of subscribers utilizing care at lower facilities is encouraging considering the financial challenges the scheme is facing as a result of high and increasing claims cost.

The relatively low adjustment made from the tertiary/teaching hospitals claims suggests that the paper-based review system have inadequate capacity to review claims from these facilities which are mainly inpatient claims; thus, require claims reviewers with specialised domain knowledge, for example, clinicians. This result also contradicts a study by Park et al. [9]. The lowest adjustment rate from the pharmacies claims is expected because the rules governing their claims preparation is quite simple. What might have accounted for the adjustments are errors in the quantity and price of medicines supplied to the subscribers.

Unlike the paper-based claims review, the electronic-based review made more adjustments from the tertiary/teaching hospitals claims and low adjustments from the regional hospitals claims. This finding is logical since claims from the higher level healthcare facilities are mostly for inpatient services, which requires stringent claims preparation as explained earlier. As a result, most of these claims are more likely to be flagged for adjustments due to errors, omissions and duplications. The finding corroborates a study by Park et al., where higher level of care facilities recorded higher rate of adjustments [9].

### Limitations

Disaggregated data on claims adjustments for outpatient cost and inpatient cost in the database were not accessible; thus, the study could not assess adjustment rate by type of healthcare services for the two reviews. Likewise, the number of claims flagged by both claims reviews and the associated reasons were also not readily available, making it impossible for the study to analyse rejection ratios of the two review systems. However, the analysis of total adjustments for each type of healthcare facility in the study provides overview of the two reviews’ capacity to reduce claims cost to the scheme.

## Conclusions

The electronic-based claims review significantly adjusted higher amounts from healthcare provider claims than the paper-based review, indicating that it has a higher ability to detect spurious claims. Scaling up the electronic-based review to cover claims from all accredited healthcare providers could reduce cost to the scheme and ensure long term financial viability. Nonetheless, there would be a need for the clinical rules of the electronic-based claims to be strengthened to make it more robust and efficient. Introduction of data mining into the electronic claims review would also be necessary for extracting and analysing useful information from thousands of claims received every month to make informed decisions.

## Additional files

Additional file 1: Table 4. Difference in adjustment rate by healthcare facility type (paper-based claims review). (DOCX 18 kb)
Additional file 2: Table 5. Difference in adjustment rate by healthcare facility type (electronic-based claims review). (DOCX 18 kb)
Additional file 3: Table 6. Mean cost adjustment rate between the paper- and electronic-based reviews. (DOCX 17 kb)

## Abbreviations

ANOVA: Analysis of Variance
CPC: Claims Processing Centre
ERC: Ethical Review Committee
G-DRG: Ghana Diagnosis Related Groupings
GHS: Ghana Cedis
GLM: General Linear Model
KKD: Knowledge Discovery from Databases
NCE: Normal Curve Equivalence
NHIA: National Health Insurance Authority
NHIS: National Health Insurance Scheme
USD: United States Dollar
