# Supplementary material for: Reducing medical claims cost to Ghana’s National Health Insurance scheme: a cross-sectional comparative assessment of the paper- and electronic-based claims reviews
Source: BMC Health Serv Res. 2017 Feb 6;17:115. doi: 10.1186/s12913-017-2054-1 (PMC5294897; doi:10.1186/s12913-017-2054-1)
Supplement: Additional file 1: Table 4. — Difference in adjustment rate by healthcare facility type (paper-based claims review). (DOCX 18 kb) [file 12913_2017_2054_MOESM1_ESM.docx]

**Table 4: Difference in adjustment rate by healthcare facility type (paper-based claims review)**

| Source | SS | df MS |  | Number of obs | = 799 |
| --- | --- | --- | --- | --- | --- |
|  |  |  |  | F( 4, 794) | = 0.62 |
| Model | 437.59 | 4 109.39 |  | Prob > F | = 0.6484 |
| Residual | 140117.57 | 794 176.47 |  | R-squared | = 0.0031 |
|  |  |  |  | Adj R-squared | = -0.0019 |
| Total | 140555.17 | 798 176.13 |  | Root MSE | = 13.284 |
|  |  |  |  |  |  |
| Healthcare facility type | Coef. | Std. Err. t | P>t | [95% Conf. | Interval] |
| Health centres & clinics | .31 | 2.12 0.15 | 0.882 | -3.86 | 4.49 |
| Polyclinics & district hosp. | -.39 | 2.16 -0.18 | 0.855 | -4.63 | 3.84 |
| Regional hosp. | 4.29 | 3.80 1.13 | 0.259 | -3.17 | 11.76 |
| Tertiary/teaching hosp. | -1.41 | 3.65 -0.39 | 0.698 | -8.60 | 5.763 |
| _cons | 11.71 | 2.02 5.78 | 0.000 | 7.738 | 15.69 |

Reference: Pharmacies
