# Supplementary material for: Reducing medical claims cost to Ghana’s National Health Insurance scheme: a cross-sectional comparative assessment of the paper- and electronic-based claims reviews
Source: BMC Health Serv Res. 2017 Feb 6;17:115. doi: 10.1186/s12913-017-2054-1 (PMC5294897; doi:10.1186/s12913-017-2054-1)
Supplement: Additional file 2: Table 5. — Difference in adjustment rate by healthcare facility type (electronic-based claims review). (DOCX 18 kb) [file 12913_2017_2054_MOESM2_ESM.docx]

**Table 5: Difference in adjustment rate by healthcare facility type (electronic-based claims review)**

| Source | SS | df MS |  | Number of obs | = 85 |
| --- | --- | --- | --- | --- | --- |
|  |  |  |  | F( 2, 82) | = 2.82 |
| Model | 392.72 | 2 196.36 |  | Prob > F | = 0.0656 |
| Residual | 5715.29 | 82 69.69 |  | R-squared | = 0.0643 |
|  |  |  |  | Adj R-squared | = 0.0415 |
| Total | 6108.01 | 84 72.71 |  | Root MSE | = 8.3486 |
|  |  |  |  |  |  |
| Healthcare facility type | Coef. | Std. Err. t | P>t | [95% Conf. | Interval] |
| Regional hosp. | -3.95 | 2.82 -1.40 | 0.165 | -9.58 | 1.66 |
| Tertiary/teaching hosp. | 5.80 | 3.31 1.75 | 0.083 | -.78 | 12.40 |
| _cons | 28.94 | 1.01 28.59 | 0.000 | 26.92 | 30.95 |

Reference: District hosp.
