# Supplementary material for: Reducing medical claims cost to Ghana’s National Health Insurance scheme: a cross-sectional comparative assessment of the paper- and electronic-based claims reviews
Source: BMC Health Serv Res. 2017 Feb 6;17:115. doi: 10.1186/s12913-017-2054-1 (PMC5294897; doi:10.1186/s12913-017-2054-1)
Supplement: Additional file 3: Table 6. — Mean cost adjustment rate between the paper- and electronic-based reviews. (DOCX 17 kb) [file 12913_2017_2054_MOESM3_ESM.docx]

**Table 6: Mean cost adjustment rate between the paper- and electronic-based reviews**

| Group | Obs | Mean | Std. Err. | Std. Dev. | [95% Conf. | Interval] |
| --- | --- | --- | --- | --- | --- | --- |
| Paper-based reviews | 799 | 11.78 | .46 | 13.27 | 10.85 | 12.70 |
| Electronic-based reviews | 85 | 28.95 | .92 | 8.52 | 27.11 | 30.79 |
| combined | 884 | 13.43 | .46 | 13.84 | 12.51 | 14.34 |
| diff |  | -17.17 | 1.47 |  | -20.059 | -14.28 |

t(882)=-11.6724, p<0.001
